# No significant improvement of cardiovascular disease risk indicators by a lifestyle intervention in people with Familial Hypercholesterolemia compared to usual care: results of a randomised controlled trial

**DOI:** 10.1186/1756-0500-5-181

**Published:** 2012-07-04

**Authors:** Karen Broekhuizen, Mireille NM van Poppel, Lando L Koppes, Iris Kindt, Johannes Brug, Willem van Mechelen

**Affiliations:** 1 Department of Public and Occupational Health, EMGO+Institute for Health and Care Research, VU University Medical Center, room G0.30, P.O. Box 7057 , 1007MB, Amsterdam, The Netherlands; 2 TNO Quality of Life, Division Work and Employment, Hoofddorp, The Netherlands; 3 Foundation for the Identification of Persons with Inherited Hypercholesterolemia (StOEH), Amsterdam, The Netherlands; 4 Department of Epidemiology and Biostatistics, EMGO+Institute for Health and Care Research, VU University Medical Centre, Amsterdam, The Netherlands

## Abstract

**Background:**

People with Familial Hypercholesterolemia (FH) may benefit from lifestyle changes supporting their primary treatment of dyslipidaemia. This project evaluated the efficacy of an individualised tailored lifestyle intervention on lipids (low density lipoprotein cholesterol (LDL-C), high density lipoprotein cholesterol (HDL-C), total cholesterol (TC) and triglycerides), systolic blood pressure, glucose, body mass index (BMI) and waist circumference in people with FH.

**Methods:**

Adults with FH (n = 340), recruited from a Dutch cascade screening program, were randomly assigned to either a control group or an intervention group. The personalised intervention consisted of web-based tailored lifestyle advice and personal counselling. The control group received care as usual. Lipids, systolic blood pressure, glucose, BMI, and waist circumference were measured at baseline and after 12 months. Regression analyses were conducted to examine differences between both groups.

**Results:**

After 12 months, no significant between-group differences of cardiovascular disease (CVD) risk indicators were observed. LDL-C levels had decreased in both the intervention and control group. This difference between intervention and control group was not statistically significant.

**Conclusions:**

This project suggests that an individually tailored lifestyle intervention did not have an additional effect in improving CVD risk indicators among people with FH. The cumulative effect of many small improvements in all indicators on long term CVD risk remains to be assessed in future studies.

**Trial registration:**

NTR1899 at ww.trialregister.nl

## Background

Familial hypercholesterolemia (FH) is an autosomal dominant disorder of the lipoprotein metabolism. Due to a defect of the low density lipoprotein (LDL) receptor gene, plasma concentrations of LDL cholesterol (LDL-C) are elevated [1]. In the Netherlands, approximately one in 500 people is affected with the heterozygous type of FH [2]. Elevated serum LDL-C and therefore FH is associated with an elevated risk of premature cardiovascular disease (CVD)[3], which is the disease with the highest burden in disability adjusted life years in the Netherlands [4]. If elevated LDL-C is not diagnosed and treated, the cumulative risk of developing coronary artery disease (CAD) by the age of 60 years is over 60% for men, and over 30% for women [5]. Large primary and secondary prevention trials with statins have clearly demonstrated the benefit of reducing LDL-C in subjects with high LDL-C [6,7]. Also, Versmissen and colleagues showed an overall risk reduction in a large cohort (n = 2146) of people with FH that used statins [8]. Still, significant CVD risk persists despite effective LDL-C lowering statin treatment [9].

Apparently, lifestyle factors can play an important role in moderating the course of this disorder [10,11], as is underlined by the EUROASPIRE III survey, conducted in 2006–2007 in 22 European countries. This survey showed a high prevalence of unhealthy lifestyles among CVD patients treated by cardiologists, and moreover, that use of medication was often inadequate to achieve treatment goals [12]. Results of primary prevention trials in high-risk persons and secondary prevention trials in CVD patients both show that substantial reductions in the CVD risk can be obtained through lifestyle changes [13,14]. For example, the INTERHEART study showed that eating fruit and vegetables daily, being physically active regularly and avoiding smoking were effective in reducing the risk of a myocardial infarction by 80% [15]. Estimates from a study by Hopkins suggested that a cholesterol-lowering diet could reduce LDL-C levels by up to 21% in people with heterogeneous FH [16]. Clearly, a healthy lifestyle is an aspect of the treatment of FH with benefits beyond LDL-C-lowering drugs [17]. FH treatment should not merely focus on LDL-C, but also on a larger spectrum of risk factors [18]. We therefore assumed that raised awareness of the actual CVD risk, improved lifestyle behaviours and improved compliance to statin therapy is a promising strategy in reducing CVD risk in people with FH.

In the PRO-FIT project, we developed an individually tailored lifestyle intervention aimed at a CVD risk reduction in individuals with FH. At first, we investigated the efficacy of the intervention on smoking, physical activity, dietary intake and compliance to statin therapy (Broekhuizen K, Msc, unpublished data, 2011). In this paper, we report the efficacy on biological CVD risk indicators: lipids (LDL-C, HDL-C, TC and triglycerides), systolic blood pressure, glucose, body mass index (BMI) and waist circumference.

## Methods

### Design and participants

A randomised controlled trial was conducted with measurements at baseline and at 12 months post-baseline. Participants diagnosed with FH from January 1^st^ 2007 to April 15^th^ 2009, aged from 18 to 70 years and with a LDL-C level > 75^th^ percentile (age and gender specific) were recruited from the national cascade screening programme of the Foundation for the Identification of Persons with Inherited Hypercholesterolemia (StOEH). Access to internet, sufficient fluency in Dutch and residency <150 km radius from Amsterdam were additional eligibility criteria. Invitation brochures were send to 986 people, of whom 321 (32%) responded and agreed to participate. An additional 23 participants were recruited through brochures that were distributed among family members of participants, meeting the same eligibility criteria. The recruitment period lasted 6 months and resulted in 340 participants. Three hundred and fifteen participants (93%) attended the baseline and follow-up measurements.

**Figure 1:**
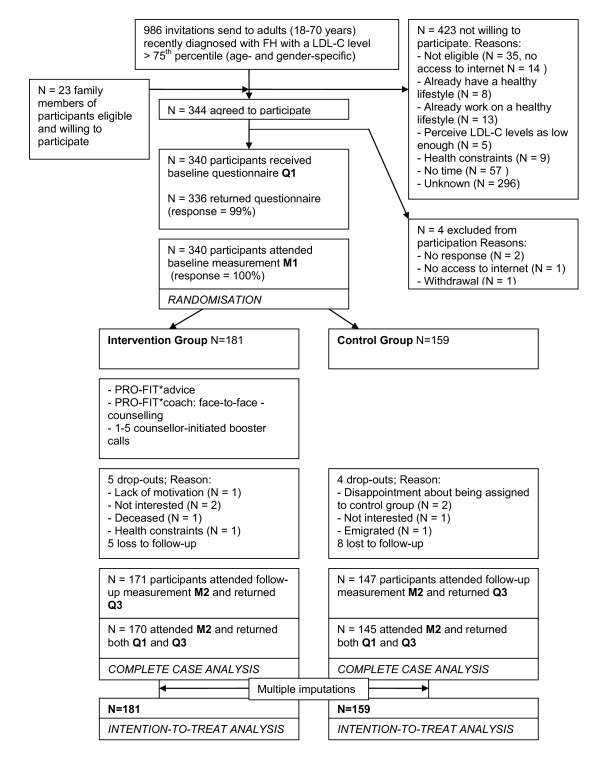
**Recruitment, participant and retention flow.** People diagnosed with FH from January 1^st^ 2007 to April 15^th^ 2009, aged from 18 to 70 years, with a LDL-C level > 75^th^ percentile (age and gender specific), with access to internet, sufficient fluency in Dutch and residency < 150 km radius from Amsterdam were considered as eligible for participation and recruited from the national cascade screening programme of the Foundation for the Identification of Persons with Inherited Hypercholesterolemia (StOEH). Invitation brochures were send to 986 people, of whom 321 (32%) responded and agreed to participate. An additional 23 participants were recruited through brochures that were distributed among family members of participants, meeting the same eligibility criteria. The recruitment period lasted 6 months and resulted in 340 participants. Three hundred and fifteen participants (93%) attended the baseline and follow-up measurements. Missing data on lipids (LDL-C, HDL-C, TC and triglycerides), systolic blood pressure, glucose, BMI and waist circumference were imputed using multiple imputations, allowing an intention-to-treat analysis based on 340 participants.

Details on the participant flow can be found in Figure 1. The content of this paper was guided by the recommendations for reporting randomised controlled trials of the CONSORT (Consolidated Standards of Reporting Trials) statement [19]. The ethical principles of the Helsinki Declaration were followed and the PRO-FIT project was approved by the Medical Ethics Committee of the VU University Medical Centre. All participants gave written informed consent.

### Procedure

Participants were randomly assigned to either the no-intervention control group (n = 159) or the intervention group (n = 181) through a stratified computerised randomisation procedure using Microsoft© Office Access 2003 software. Randomisation was concealed. At first, participants were stratified according to cholesterol lowering medication use (yes/no), assuming that medication use implicates treatment by a general practitioner and/or medical specialist, who could have already given advice on lifestyle behaviour. In addition, we expected that a decrease in LDL-C because of the intervention would be smaller if a participant already used medication. Family members of the same household were clustered and subsequently randomised as a cluster to prevent contamination of the intervention effect due to spill over of communication about the intervention among family members.

### Theoretical framework

**Figure 2:**
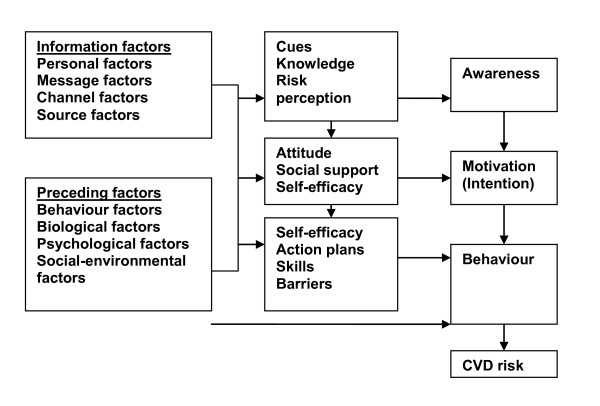
**The I-Change model 2.0.** The I-Change model assumes that the behavioural change process can be distinguished in three phases: 1) Awareness, 2) Motivation and 3) Action. Hypothetically, due to gained knowledge and awareness of one’s CVD risk, a participant will become motivated to change lifestyle behaviour(s), and subsequently, implementation intentions and action plans will be formed to actually achieve (maintenance of) behavioural change. In addition, it is assumed that this will eventually lead to a reduction in CVD risk.

The intervention of the PRO-FIT project was developed according to the integrated model for exploring motivational and behavioural change, the I-Change model (2.0) [20,21]. Briefly, it assumes that the behavioural change process can be distinguished in three phases: 1) Awareness, 2) Motivation and 3) Action. Hypothetically, due to gained knowledge and awareness of one’s CVD risk, a participant will become motivated to change lifestyle behaviour(s), and subsequently, implementation intentions and action plans will be formed to actually achieve (maintenance of) behavioural change. In addition, it is assumed that this will eventually lead to a reduction in CVD risk. The assumed pathway is illustrated in the I-Change model (2.0) in Figure 2.

### Intervention

The intervention consisted of a personalised health counselling intervention; a combination of computer-generated tailored web-based advice (PRO-FIT*advice) and face-to-face counselling complemented with telephone booster sessions (PRO-FIT*coach). The goal was to: 1) improve awareness of the cardiovascular disease risk through an increase of specific knowledge, cues to action and change in risk perception, 2) improve motivation with respect to healthy behaviour through an increase of specific knowledge and a change in attitude, self-efficacy and social influences, 3) adopt and maintain a healthier lifestyle, with regard to physical activity, saturated fat intake, fruit and vegetables intake, smoking and compliance to statin therapy, and 4) lower the level of LDL-C and other biological CVD risk indicators and thereby reducing the CVD risk.

The intervention has been described in detail elsewhere [21]. Briefly, participants were encouraged to visit a web link referring to the project website, where generic online CVD risk information was presented, containing feedback on CVD risk behaviours and their contribution to overall CVD risk, as well as information on the changeability of these behaviours and cues on how to change behaviours. Thereafter, participants could log on to a personal PRO-FIT*advice account, consisting of six tailored advice modules on smoking, physical activity, saturated fat intake, fruit intake, vegetables intake and compliance to statin therapy. On-screen computer-generated personalised feedback was tailored to personal performance level (current lifestyle behaviour), awareness of one’s own performance, as well as personal motivation to change, outcome expectations, attitude and self-efficacy. Personalised feedback on compliance to statin therapy was tailored to knowledge and personal beliefs about (the effect of) statin therapy, potential side effects of the prescribed drug and current compliance.

Subsequently, a month later, the participant and the personal coach further established the level of the participant’s knowledge/awareness about FH and cardiovascular risk factors. Furthermore, the assessment(s) and advice(s) within the participant’s personal PRO-FIT*advice account were discussed and ambivalence and barriers related to the recommended behaviour changes were explored based on Motivational Interviewing (MI) techniques [22]. Further, one to five counsellor-initiated booster telephone sessions were performed during a period of 9 months to encourage the participant’s behavioural changes and to provide further brief motivational interviewing to encourage the planned behavioural changes.

The control group received care as usual.

### Measurements

In this project, lipids (LDL-C, HDL-C, TC and triglycerides), systolic blood pressure, glucose, BMI and waist circumference were defined as CVD risk indicators, also known as classical CVD risk factors, as reported by the Adult Treatment Panel (ATP) III of the NCEP, that formulated an evidence-based set of guidelines of cholesterol management for the general population [23]. These classical risk factors also contribute to the CVD risk in people with FH [24].

All CVD risk indicators were measured at the participants’ homes. LDL-C, HDL-C and TC, triglycerides and glucose were measured with fasting finger stick samples analysed on a Cholestech LDX desktop analyser (Cholestech, Hayward, USA). This portable analyser is capable of providing a lipid profile and glucose in approximately 5 minutes. The reproducibility and precision of lipids measurement by the LDX analyser are within the guidelines of the NCEP [25,26]. The Cholestech LDX analyser has been validated for point-of-care lipid measurements in clinical practice [27]. Systolic and diastolic blood pressure (in mmHg) was measured twice with a fully automated blood pressure monitor (type: Omron M5-I). The mean value of the two measurements was computed.

Body height (in cm) was measured on bare feet with a portable device with a wide measuring slide and a heel plate. Calibrated scales were used to determine body weight (in kg) while participants wore light clothing only (e.g. underwear). Both body weight and height were measured twice, and the mean value of the two measurements was used to calculate BMI. Waist circumference (in cm) was measured twice with a measurement tape to the nearest 0.10 cm, at the midpoint between the lower border of the ribs and the upper border of the pelvis. The mean value of the two measurements was computed.

A process evaluation was carried out, taking into account the process elements reach, dose (delivered and received) and fidelity [28]. In short, reach (the number of people included in the project, as well as their representativeness for the study population and non-participants) was assessed by consulting the StOEH/PRO-FIT client database. The dose of all delivered elements of the intervention was assessed by logs that were kept by the coaches and the project database. Dose received, i.e. the way participants used PRO-FIT*advice (% of participants that logged on, number of modules finished), was assessed by means of log on rates and website use data. Whether face-to-face counseling sessions were implemented as planned according to Motivational Interviewing (MI) guidelines (fidelity) was assessed by two MI experts, following the MI Treatment Integrity code 3.1.1 [29]. For this assessment, a random sample of 20 audio taped counselling sessions (10 sessions of each lifestyle coach; approximately 10% of all sessions) was drawn. A verbatim transcript [30] of each drawn session was evaluated and resulted in two scores: a global score, capturing an overall impression of the conversation on a 5-point Likert scale on the following 5 dimensions: Evocation, Collaboration, Autonomy/Support, Direction and Empathy. In addition, specific behaviours of the lifestyle coach, such as the number of open/closed questions and simple/complex reflections (reflective statements made by the counsellor in response to participant, without/with additional meaning or emphasis to what the participant has said) were counted. Counselling sessions were considered MI if the average of global scores was ≥ 3.5, reflection to question ratio was in favour of reflection, >50% of the questions were open questions, >40% of the reflections were complex reflections and >90% of all utterances was MI-adherent [29].

### Statistical analyses

Potential baseline differences were checked between intervention and control group, regarding gender, age, education, BMI, medication use and LDL-C. In case of baseline differences between intervention and control group, the concerned covariate was included in the analyses. In addition, differences between dropouts and participants regarding the above-mentioned baseline characteristics were tested by linear regression analyses.

Primary, a complete case analysis was conducted at the participant level, restricted to those who attended baseline and follow-up measurements. These numbers vary for different outcome measures. Subsequently, an intention-to-treat analysis was conducted, involving all participants who were randomly assigned (n = 340). Missing data on lipids (LDL-C, HDL-C, TC and triglycerides), systolic blood pressure, glucose, BMI and waist circumference were imputed using multiple imputations. Five different datasets were created in SPSS (version 18.0) using Fully Conditional Specification and Predictive Mean Matching procedures. All available data on the above-mentioned outcomes, as well as on group allocation, gender, age, education, BMI, medication use and LDL-C were included in the imputation model. Thereafter the multiple datasets were analysed as described below, using SPSS (version 18.0). Pooled estimates were computed following the rules as described by Rubin [31]. As no major differences were found, only the results of the complete case analysis are presented.

In order to investigate whether this intervention had an effect on lipids (LDL-C, HDL-C, TC and triglycerides), systolic blood pressure, glucose, BMI and waist circumference, linear regression analyses were conducted to detect between group differences after 12 months (two-sided; significance level 0.05). The post-test scores were regressed on study group and baseline measure of the outcome variable.

## Results

### Baseline characteristics of participants

In Figure 1 the recruitment, participant and retention flow is presented. As can be seen from Table 1, the participants were equally distributed with regard to gender. Overall, a mainly middle aged, medium to highly educated, fairly overweight and the majority has an elevated LDL-C and used cholesterol-lowering medication. Baseline differences between the control and intervention group were found for BMI (β = −1.10; CI −2.17- −0.04). As a consequence, this variable was included in the regression analyses as a potential confounder. No differences were found between dropouts and participants regarding the baseline characteristics.

**Table 1:** Baseline characteristics of the control and intervention group

	Control group	Intervention group
Gender (% female; N)	56.3; N = 159	57.1; N = 181
Age (years, mean ± SD; N)	45.9 (13.0); N = 159	44.7 (12.9); N = 181
Education (%; N) low medium high	3.6 62.8 33.6; N = 137	3.1 58.2 38.7; N = 163
BMI (kg/m^2^, mean ± SD; N)	**27.1 (5.3); N = 159**	**26.0 (4.7); N = 181**
Medication use (% yes; N)	69.6; N = 159	68.8; N = 181
LDL-C (mmol/l, mean ± SD; N)	3.7 (1.2); N = 130	3.7 (1.3); N = 146

N = sample size; SD = standard deviation; BMI = body mass index; Significant differences between control and intervention group (P < 0.05) are printed in bold font.

### Effect on biological risk indicators

After 12 months, LDL-C had decreased in both the intervention and control group (see Table 2). No significant between-group difference was found, as well as for HDL-C, TC, triglycerides, systolic blood pressure, glucose, BMI and waist circumference. Both groups showed no major changes in HDL and a slight decrease of TC and glucose. A minor increase of triglycerides was seen in the intervention group, in contrast to a decrease in the control group. In the control group, no change in BMI was observed after 12 months, compared to a decrease in the intervention group. Waist circumference did not change in the control group and decreased in the intervention group.

**Table 2:** Biological CVD risk indicators at baseline and follow-up and intervention effects from linear regression analyses, based on a complete-case analysis^*^

	Control group	Intervention group	beta	95% CI
LDL-C (mmol/l, mean ± SD) Baseline 12 months Difference	N = 105 3.7 (1.2) 3.6 (1.2) −0.1	N = 128 3.6 (1.3) 3.5 (1.1) −0.1	−0.20	−0.40-0.03
HDL-C (mmol/l, mean ± SD) Baseline 12 months Difference	N = 143 1.2 (0.4) 1.2 (0.4) 0	N = 169 1.2 (0.4) 1.2 (0.4) 0	0.02	−0.04-0.08
TC (mmol/l, mean ± SD) Baseline 12 months Difference	N = 146 5.2 (1.2) 5.1 (1.2) −0.1	N = 169 5.3 (1.4) 5.2 (1.2) −0.1	−0.04	−0.25-0.18
Triglycerides (mmol/l, mean ± SD) Baseline 12 months Difference	N = 110 1.3 (0.7) 1.2 (0.6) −0.1	N = 128 1.2 (0.6) 1.3 (0.7) +0.1	0.08	−0.08-0.23
Systolic blood pressure (mmHg, mean ± SD) Baseline 12 months Difference	N = 143 126.3 (15.7) 125.2 (14.4) −1.1	N = 169 123.0 (14.4) 123.0 (14.1) 0	0.003	−2.28-2.28
Glucose (mmol/l, mean ± SD) Baseline 12 months Difference	N = 145 4.9 (1.0) 4.8 (0.8) −0.1	N = 169 4.9 (0.8) 4.7 (0.7) −0.2	−0.06	−0.19-0.07
BMI (kg/m^2^, mean ± SD) Baseline 12 months Difference	N = 147 27.1 (5.4) 27.1 (5.2) 0	N = 167 25.9 (4.5) 25.8 (4.4) −0.1	−0.18	−0.43-0.07
Waist circumference (cm, mean ± SD) Baseline 12 months Difference	N = 146 89.9 (14.5) 89.9 (14.3) 0	N = 165 86.4 (11.9) 86.1 (11.5) −0.3	−0.54	−1.45-0.40

^*^Differences between control and intervention group after 12 months are tested through linear regression analyses, controlled for baseline values and baseline BMI. Beta = unstandardised regression coefficient; N = sample size; SD = standard deviation; 95% CI = 95% confidence interval as effect indicator from linear regression analyses; Means presented are from unadjusted analyses; Significant differences between control and intervention group (P < 0.05) printed in bold font. Only the results of the complete case analysis are presented, since no major differences were found between intention-to-treat analysis and complete case analysis.

### Process

A 34% (n = 181) representative proportion of the intended intervention group was reached during the recruitment phase; participants did not differ from non-participants (n = 623) in the StOEH client database on age, gender and LDL-C levels. Of the participants, 95% received a PRO-FIT*advice log on account, of which 49% actually logged on and completed at least one advice module. Nearly all participants received a face-to-face counseling session and on average, 4.2 telephone booster calls were delivered. None of the face-to-face sessions were implemented according to MI guidelines.

## Discussion

In this paper, the efficacy of an individually tailored lifestyle intervention on lipids (LDL-C, HDL-C, TC and triglycerides), systolic blood pressure, glucose, BMI and waist circumference in people with FH was investigated. After 12 months, LDL-C levels had decreased in both the intervention and control group. This difference between intervention and control group was not statistically significant. Furthermore, the LDL-C concentrations in both groups did not result in reaching the recommended treatment target concentration of ≤2.5 mmol/l for most participants [32]. Based on a comparable population, Huijgen and colleagues also concluded that only a minority of the medication users reaches LDL-C treatment targets within two years after screening [33].

Overall, we also did not observe any significant intervention effects on HDL-C, TC, triglycerides, systolic blood pressure, glucose, BMI and waist circumference. However, it may be that the collective contribution of all these small improvements together is cumulative, and larger than the CVD risk reduction associated with a single risk indicator. But since to date, no CVD risk prediction tool is available for FH populations, it is impossible to have an accurate estimate of the CVD risk reduction from all the small improvements together. Such a CVD risk prediction tool would be beneficial for the interpretation of our results. Moreover, it would be possible to identify people with severely increased CVD risk. Several CVD risk estimates are available, such as the risk assessment tool that uses data from the Framingham Heart Study to estimate 10-year risk for hard coronary heart disease outcomes [34]. However, these tools are based on calculations in a non-FH reference population, and it is known that classical risk factors in a FH population do not necessarily play the same role with the same intensity [24]. Civeira proposed a risk assessment tool, dividing people with FH in three risk categories: low-moderate-high 10 year CVD mortality risk [17]. However, lipoprotein-A and carotid intima media thickness, defined as major risk factors by Civeira, were not assessed in this project.

Participants who were not on statin treatment had notably higher LDL-C and TC levels at baseline, and according to post-hoc analyses, reductions in LDL-C and TC concentrations were most obvious among participants who used no statins (data not shown). Clearly, a reduction in lipids levels is most expected among this subsample, since more reduction of lipid levels can be achieved. However, due to the small subsample (n = 72), this finding should be interpreted with great caution.

We did not find any significant intervention effects on all targeted lifestyle behaviours in the PRO-FIT project (data not shown). We can not confirm nor reject whether small improvements of biological CVD indicators were caused by behavioural improvements. The lack of intervention effects on biological CVD risk indicators and lifestyle behaviours found in the PRO-FIT project are not in accordance with the latest evidence, as in a literature review, Blokstra et al. showed that multifactorial lifestyle interventions could have favorable effects among individuals with a high CVD risk: improvements in blood pressure (−2-4 mmHg), nutrition, physical activity and smoking (−25-40%) were found [14]. Studies on other high-risk populations also showed that biological changes can be achieved, though often small and not significant at a long term (> 6 months) [35,36]. However, the above-mentioned studies did not include FH subjects. In a recent review of Shafiq (2011), no differences were reported between cholesterol-lowering diet in comparison with no intervention or other dietary interventions in people with FH [37].

It may be that the intervention reach and dose received were insufficient to initiate behaviour changes and, subsequently, changes in CVD indicators. Our process evaluation indicates that participants were sufficiently exposed to the intervention. However, only half of the participants logged on at the PRO-FIT*advice website and completed at least one of the advice modules, and face-to-face counselling sessions were delivered with low MI fidelity.

More in-depth analysis showed weak and positive associations between dose and LDL-C change for all intervention components (data not shown). Due to the small sample of audio taped sessions (n = 20), the association between MI fidelity and efficacy could not be tested in this study, but previous studies showed that a better MI performance is associated with larger intervention effects [22,38]. Also, mixed evidence has been published on computer-tailored interventions addressing more than one lifestyle behaviour. It is possible that multiple-behaviour interventions may be burdensome for some individuals, and advices may be too long [39-41]. Overall, it is possible that the poor MI fidelity and dose of PRO-FIT*advice received contributed to the lack of efficacy. Probably, the provided MI workshop was not sufficient and more thorough monitoring and supervision of counselling skills during the intervention should have been built in, as it has often been reported that skills required for effective MI may take longer to develop than the 3-day MI workshop in our project [42,43].

The strengths of this project include the randomised design, which avoided study contamination that could have resulted from individual changes of participants in both intervention and control group. To our knowledge, the PRO-FIT intervention is the first to evaluate the effects of a lifestyle intervention on multiple lifestyle behaviours and CVD risk indicators among people with FH. The RCT was conducted in a sample representative for the screened FH population in the Netherlands with a small dropout rate. However, the recruitment rate of our study was only 34% and contained a self-selected sample of mainly medium-educated participants with internet access and sufficient fluency in Dutch. Although no differences on age, gender and LDL-C levels were found between participants and non-participants, our sample could have been more motivated to change lifestyle behaviour, which might limit the generalisability of our findings [44].

While various unhealthy lifestyle factors are related to the atherosclerotic process, it is the long-term exposure that leads to the clinical manifestations of cardiovascular events [45]. Vice versa, the effect of lifestyle improvements is likely to lead to CVD risk reduction only at the longer term. Inclusion of more long-term follow-up measurements in future RTCs on the efficacy of lifestyle interventions is to be recommended, since it would shed more light on possible effects on CVD risk and hard outcomes (e.g. CVD/death).

Altogether, it remains unclear how genetic and lifestyle factors interact in the FH population. FH is a monogenetic condition, though variations are found in mortality [46], suggesting that environmental factors play an important role as well. Whether the gene-environment interactions are synergistic or simply additive remains to be revealed. It would be informative to conduct a trial similar to the PRO-FIT project with the inclusion of a non-FH control group with elevated LDL-C levels. Despite the unknown interactions, the primary goal of treatment of people with FH should be considered as invariable: achieving optimal CVD risk reduction.

In conclusion, this project suggests that an individually tailored lifestyle intervention is not superior to usual care, regarding changes in LDL-C levels in people with FH. A small CVD risk reduction might result from the generally slight improvements of all the CVD risk indicators. However, in order to draw conclusions on impact of the cumulative effect of all these small improvements, and thus on the efficacy of lifestyle improvements in a FH population, more RCTs should be performed, including more long-term objective measurements (of e.g. carotid intima thickness and lipoprotein A) and long-term monitoring of CVD-related morbidity and mortality.

## Conclusions

An individually tailored lifestyle intervention did not have an additional effect in reducing LDL-C levels among people with FH. The cumulative effect of many small improvements in all CVD risk factors on long term CVD risk remains to be assessed in future studies.

## Competing interests

The authors declare that they have no competing interests.

## Authors’ contributions

KB was responsible for data analysis, interpretation and reporting, while MvP, LK, IK, JB and WvM assisted in interpreting and reporting. All authors read, edited and approved the final version of the manuscript.
